# Macrophage-Mediated Subversion of Anti-Tumour Immunity

**DOI:** 10.3390/cells8070747

**Published:** 2019-07-19

**Authors:** Valeria Quaranta, Michael C. Schmid

**Affiliations:** Department of Molecular and Clinical Cancer Medicine, University of Liverpool, Ashton Street, Liverpool L69 3GE, UK

**Keywords:** macrophage, cancer, anti-tumour immunity, immunosuppression, immune checkpoint blockers

## Abstract

Despite the incredible clinical benefits obtained by the use of immune checkpoint blockers (ICBs), resistance is still common for many types of cancer. Central for ICBs to work is activation and infiltration of cytotoxic CD8^+^ T cells following tumour-antigen recognition. However, it is now accepted that even in the case of immunogenic tumours, the effector functions of CD8^+^ T cells are highly compromised by the presence of an immunosuppressive tumour microenvironment (TME) at the tumour site. Tumour-associated macrophages (TAMs) are among the most abundant non-malignant stromal cell types within the TME and they are crucial drivers of tumour progression, metastasis and resistance to therapy. TAMs are able to regulate either directly or indirectly various aspects of tumour immunity, including T cell recruitment and functions. In this review we discuss the mechanisms by which TAMs subvert CD8^+^ T cell immune surveillance and how their targeting in combination with ICBs represents a very powerful therapeutic strategy.

## 1. Introduction

Tumours comprise multiple distinct cell types that reciprocally interact with one another. Indeed, cancer cells are able to corrupt and recruit normal cell types, referred to as cancer-associated stroma [1]. Stroma cells, including endothelial cells, fibroblasts, pericytes, lymphocytes and myeloid cells are responsible for the formation of the tumour microenvironment (TME) that is the cellular environment in which solid tumours exist. The systemic communication between cancer cells and the rich TME sustains the overall tumour growth, homeostasis, and progression and modulates the therapeutic response of tumours [2].

Immune checkpoint blockade (ICB)-based immunotherapy is designed to amplify an endogenous anti-tumour T cell response. Nowadays, checkpoint blockade, in particular the targeting of programmed cell death-1 (PD-1)/(PD-1) ligand (PD-L1) pathway, is considered one of the most promising anti-cancer treatments and enormous therapeutic benefits have been reported for its use in different cancer types [3]. Central for ICBs to work is the infiltration of cytotoxic CD8^+^ T cells at the tumour site and their initial cytolytic activation [4]. Although CD8^+^ T cell activation and infiltration has been strictly correlated with tumour antigenicity, it is now accepted that T cell mediated anti-tumour immunity is also compromised by the presence of an immunosuppressive TME at the tumour site.

Indeed, the success of anti-tumour defence is determined by the ability of CD8^+^ T cells to overcome additional barriers that they may encounter within the TME posed by tumour cells, fibrotic stroma, T regulatory (T_reg_) cells, myeloid cells, inhibitory cytokines, and other stroma components that act to mitigate the anti-tumour response [5]. Tumour-associated macrophages (TAMs) are one of the most abundant non-malignant cell types within the TME and they participate in all the steps of tumorigenesis from tumour development to metastasis formation and resistance to therapy [6,7]. Recently, the role of TAMs has been also associated with modulation of anti-tumour immunity. In this review we describe our current understanding of the mechanisms by which TAMs induce CD8^+^ T cell immunosuppression and how their targeting is now evaluated to increase the impact of ICB therapies.

## 2. Regulation of CD8^+^ T Cell Response: Development of Immune Check-Point Blockers

**Figure 1:**
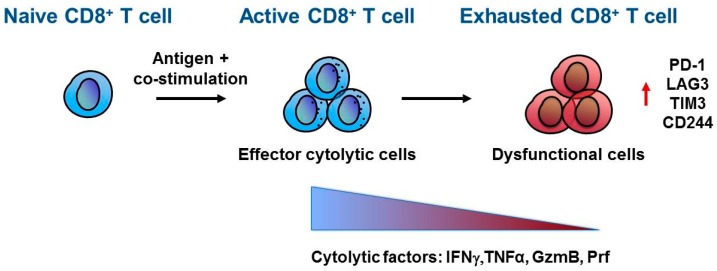
CD8^+^ T cell exhaustion. Naïve CD8^+^ T cells differentiate and activate into effector CD8^+^ T cells upon antigen recognition and co-stimulation mediated by antigen presenting cells (APC). Once at the tumour site, active CD8^+^ T cells recognize cancer cells by T cell receptor (TCR)–tumour antigen interaction and direct their effector anti-tumour activity through secretion of cytokines and lytic molecules such as interferon γ (IFNγ), tumor necrosis factor α (TNFα), granzyme B (GzmB) and perforin (Prf). However, within the tumour microenvironment, CD8^+^ T cells can acquire a dysfunctional or exhausted phenotype characterized by a progressive loss in cytolytic factors and increased expression of inhibitory receptors, including Programmed cell death-1 (PD-1), lymphocyte-activation gene 3 (*LAG3*), T-cell immunoglobulin and mucin-domain containing 3 (TIM-3), and CD244.

During an effective immune response, naïve CD8^+^ T cells rapidly differentiate into effector T cells with unique metabolic, functional and migratory properties that promote cellular proliferation and infiltration into peripheral tissues. Following antigen recognition, T effector cells express cytokines and lytic molecules that mediate specific killing of pathogenic or transformed cells [8]. Regulation of the CD8^+^ T cell response is a complex process consisting of both stimulatory and inhibitory cell-intrinsic signalling pathways. The process of CD8^+^ T cell activation not only leads to initiation of T cell proliferation and differentiation, but also involves the induction of inhibitory pathways that can attenuate and terminate T cell function (Figure 1). The stimulation of CD8^+^ T cell inhibitory pathways is a physiological response to limit CD8^+^ T activity and it prevents the insurgence of autoimmune diseases [9]. In the same way, tumours can exploit CD8^+^ T cell inhibitory pathways for their own benefits, thereby promoting tumour escape from immune surveillance and eradication.

Cytotoxic T-Lymphocyte Antigen 4 (CTLA-4) is expressed upon CD8^+^ T cell activation and it has very high homology to the T cell co-stimulatory receptor CD28 and, as CD28, it binds B7 molecules (CD80 and CD86) expressed on the surface of antigen-presenting cells (APC). During epitope mediated CD8^+^ T cell receptor stimulation, CTLA-4 accumulates in CD8^+^ T cells at the T cell - APC interface until it reaches a level sufficient to block co-stimulation of CD28 by competitively binding to B7 molecules, thereby abrogating CD8^+^ T cell activation and response [10]. Based on this knowledge, Allison’s group proposed that the blockade of CTLA-4 could boost the amplitude of CD8^+^ T cell activation thereby achieving a better CD8^+^ T cell-mediated tumour eradication. The group tested in several mouse experiments the use of anti-CTLA-4 antibody in combination with cancer cell killing agents in order to prime antigen release and activation of CD8^+^ T cells before CTLA-4 blockade [11,12]. These and similar findings inspired the development and the successful Food and Drug Administration (FDA) approval of Ipilimumab, an antibody against human CTLA-4 in 2011. Ipilimumab considerably improved the overall survival in patients with metastatic melanoma [13,14], renal cell carcinoma [15], prostate cancer [16], urothelial carcinoma [17] and mammary carcinoma [18].

In 2000, PD-1 emerged as another potent inhibitory immune check-point receptor that limits the response of activated CD8^+^ T cells [19]. PD-1 has two ligands, PD-L1 (also known as B7H1 or CD274), which is broadly expressed by many cell types mainly upon exposure to pro-inflammatory cytokines, and PD-L2 (also known as CD273 or B7-DC) which instead has more restricted expression on APC [20]. The function of PD-1 is different to that of CTLA-4. CTLA-4 interferes with co-stimulation of CD8^+^ T cells, is involved in the regulation of the amplitude of T cells activation at the early stages, and acts at the level of lymphatic organs. In contrast, PD-1 engagement by PD-L1 can negatively affect both CD8^+^ T cell priming at the level of lympho-nodes and CD8^+^ T cell cytotoxic activity in peripheral tissue [21]. Tumour cells express high levels of PD-L1 as an immune escape mechanism and, thus, the assessment of PD-L1 expression levels in tumour biopsies is often used as a predictive marker for therapeutic response to PD-L1/PD-1 therapy [4]. However, PD-L1 expression by stroma cells, including myeloid cells, also contributes to CD8^+^ T cell suppression, suggesting that both sources of PD-L1 expression should be considered as predictive marker for PD-1/PD-L1 therapy response [22].

CD8^+^ T cells repetitively recognize cognate antigens as the tumour develops and form metastases. Triggering of T cell antigen receptor (TCR) results in the production of inflammatory cytokines, including interferon γ (IFNγ), which is the main stimulator of PD-L1 expression in targeted cells. Chronic PD-L1 engagement by PD-1 results in continuous PD-1 signalling in T cells and induction of epigenetic programs that lead to T cell exhaustion [23]. T cell exhaustion is a state of T cell dysfunction in a chronic inflamed environment. The principal characteristic of exhausted T cells is the progressive loss of effector functions, which generally occurs in a hierarchical manner and includes loss of interleukin (IL) 2, tumour necrosis factor α (TNFα), IFN**γ**, Granzyme B (GzmB) and Perforine (Prf) production. Moreover, exhausted T cells express high levels of inhibitory receptors in addition to PD-1, such as LAG-3, CD244, and TIM-3; and it has been reported that the pattern of inhibitory receptors that are co-expressed by the same CD8^+^ T cell can substantially affect the severity of dysfunction [24] (Figure 1). The phenotype acquired by dysfunctional CD8^+^ T cells affects their survival, proliferation and cytotoxic functions and although exhaustion is not a terminal state, it ultimately leads to T cell apoptosis [25].

PD-1 is, therefore, a negative regulator of a pre-existing immune response and its blockade has become relevant in cancer therapy because of its potential to reverse T cell exhaustion with subsequent stimulation of anti-tumour immunity. Furthermore, the pharmacological blockade of PD-1/PDL-1 is mainly targeted towards PD-L1 expressing cancer cells, with less toxicity for patients in comparison to anti-CTLA-4 inhibitory antibody therapies, as has been reported in the case of advanced melanoma [26].

Antibodies targeting the PD-1/PD-L1 axis have shown clinical durable responses in multiple tumour types. The first anti-PD-1 inhibitory antibodies (Pembrolizumab and Nivolumab) received FDA approval in 2014 and since then different types of anti-PD-1/PD-L1 inhibitory antibodies are under development and/or have been approved for treatment of melanoma [27], metastatic non-small cell lung cancer [28], head and neck cancer [29], Hodgkin’s lymphoma [30] and gastroesophageal, bladder and urothelial cancers [31,32]. Also, CTLA-4 and PD-1 regulate distinct inhibitory pathways and have non-overlapping mechanisms of action [21], thus suggesting that the use of combinatorial treatments could improve the efficacy of a single treatment [33]. Clinical trials have reported increased survival advantages after the combinatorial treatment in more than 80% of advanced melanoma patients with almost half of the patients showing tumour reduction [34].

## 3. Obstacles to Anti-Tumour Immunity

Despite the enormous clinical benefits given by the use of ICBs, only patients with certain tumour types, particularly melanoma, non-small-cell lung and renal cell cancers, respond positively to this kind of therapy. In other cancer types, including pancreatic, colorectal and ovarian cancer, patients are largely refractory or only a small fraction of patients shows a positive response [35]. It became clear that the presence of an active CD8^+^ T cell response within the tumour is the key factor for prediction of anti- PD-1/PD-L1 therapeutic efficacy [36,37]. The induction of CD8^+^ T cell-mediated anti-tumour activity critically depends on tumour- mutational load and tumour- associated neo-antigens. However, it is now accepted that in addition to tumour-intrinsic factors (Reviewed in [38]) anti-tumour immune defence, and in consequence the therapeutic activity of ICB, are strictly correlated with the presence of a complex immunosuppressive network within the TME [39]. Moreover, in many solid cancer types the physical exclusion of CD8^+^ T cells from the tumour site has been demonstrated to be a critical limiting factor for ICB approaches. A dense fibrotic stroma, hypoxia, and abnormal blood vessel architecture can impede the efficient infiltration of CD8^+^ T cells into the tumour thereby prohibiting CD8^+^ T cell–cancer cell interaction. In this scenario, CD8^+^ T cells are no longer capable to physically interact with and kill cancer cells [40,41,42,43,44]. Thus, overcoming the physical barrier restrictions imposed by the TME is necessary for successful ICB therapies.

In the light of these observations, the identification of key immunosuppressive factors within the TME and their combined targeting with ICB provides an attractive treatment strategy to enhance the efficacy of ICB therapies in cancer.

## 4. Macrophage Origin and Characterization

Macrophages are phagocytic cells and they constitute the first line of defence of our immune system against invading agents. Macrophages express surface receptors that allow them to recognize danger signals that are not normally found in healthy tissue. For example, scavenger receptors are responsible for binding apoptotic cells; pattern recognition receptors (PRRs) are able to recognize signals associated with invading pathogens, foreign substances, and dead or dying cells [45]. Macrophages also express numerous cytokines, chemokines, growth factors, and proteolytic enzymes and they are considered as key regulators of tissue homeostasis and repair. Moreover, macrophages play a central role in developmental processes such as vascular and neuronal patterning, as well as in pathophysiological responses, like inflammation and organ healing regeneration [46]. Generally macrophages can be distinguished by their expression of surface markers such as EMR1 (homologous of murine F4/80), CD11b, CD18 (also known as MAC1), CD68 and Fc receptors [47].

Macrophages originate from three different developmental pathways. All tissue embryonic macrophages derive from macrophage precursors in the yolk sac and fetal liver. During adulthood, fetal macrophages are replaced gradually by macrophages derived from bone marrow hematopoietic stem cell (HSCs) [48]. Some types of tissue resident macrophages, including bone osteoclasts, epidermal Langerhans cells, lung alveolar macrophages, microglia and liver Kupffer cells develop from embryonic macrophages and persist in adult tissues independently of replenishment by Ly6C^high^ monocytes originated from HSCs during adulthood [49,50]. Instead, other types of tissue macrophages such as intestine, dermis, heart and pancreas macrophages undergo a continuous turnover in adulthood by recruitment of circulating monocytes which differentiate into macrophages upon tissue infiltration [51,52]. Infiltrating monocytes derived from HSCs are also the main source of macrophage replenishment into inflamed and remodelling tissues, and this process is driven by cytokines and chemokines, such as C-C motif chemokine ligand (CCL) 2, CCL5 and macrophage colony stimulating factor-1 (CSF-1) [53]. There are different markers to identify monocyte-macrophages diversity in human and mouse. In human, circulating monocytes, which originate from the bone marrow, can be classified in two subsets: CD14^+^ CD16^neg^ ‘inflammatory’ or ‘classical’ and CD14^+^ CD16^+^ ‘patrolling’ or ‘non-classical’ monocytes. In the same way, mouse ‘inflammatory’ monocytes are classified as CD11b^+^ Ly6C^high^ CCR2^high^ CX3CR1^low^, in contrast ‘patrolling’ monocytes are CD11b^+^ Ly6G^low^ CCR2^low^ CX3CR1^high^ [54]. Patrolling monocytes monitor the microvasculature under steady-state conditions and rarely extravasate into tissue. However they can rapidly accumulate in lung metastatic tissue and inhibit cancer cell seeding and growth by exerting anti-tumour functions through recruitment of NKs [55]. Macrophages are a population of heterogeneous and plastic cells [56]. Once resident in tissues, macrophages acquire a distinctive phenotype in response to different signals present in the immediate microenvironment. Environmental stimuli, like IFNγ or microbial products, like the lipopolysaccharide molecule (LPS), induce a classical activation of macrophages and skew them toward an M1-like or ‘classically activated’ macrophage phenotype. M1-like macrophages mediate anti-microbial and tumoricidal response by secreting inflammatory cytokines, such as TNFα, IL-12, reactive oxygen species and nitric oxide (NO), by up-regulating the expression of major histocompatibility complex (MHC II) and by promoting a T_H1_-type of response. Alternatively, if the microenvironment becomes populated by different types of cytokines and growth factors, like the T_H_ type-2 cytokines IL-4 and IL-13, macrophages are stimulated to acquire an alternative activation state, resulting in an M2-like subtype. M2-like polarized macrophages are characterized by expression of anti-inflammatory cytokines, such as IL-10, lower expression of pro-inflammatory cytokines, up-regulation of scavenger receptors, such as mannose receptors (MRC1/CD206 and CD163) and reduced ability to activate adaptive immune response. As such, M2-like macrophages may facilitate resolution of inflammation and promote tissue repair after the acute inflammation phase [46].

During cancer progression, tumours recruit a variety of immature myeloid cells that include Ly6C^+^/CCR2^+^ inflammatory monocytes and myeloid-derived suppressor cells (MDSCs) [57]. MDSCs comprise precursors of both the mononuclear monocyte/dendritic cell (DC) lineage and precursors of the neutrophils, granulocytes lineage [58]. Inflammatory monocyte and monocyte-related-mononuclear M-MDSC can infiltrate into neoplastic tissues, upon recruitment, and differentiate into tumour-associated macrophages (TAMs) [59,60]. Recruitment of circulating cells is required to maintain TAM populations. Whether tissue macrophages derived from embryonic precursors contribute to number, location and diversity of TAMs is not fully elucidated. Interestingly, several recent studies suggest that tissue resident macrophages also contribute to the TAM population in brain, lung and pancreatic cancer [61,62,63]. For example, in the case of pancreatic cancer differently originated TAMs give rise to TAMs with different phenotype and functionality: accordingly, while circulating Ly6C^high^ inflammatory monocyte-derived TAMs are more potent in antigen presentation, embryonically derived TAMs are long-lived and self-renewing cells with high expression of pro-fibrotic factors and with pro-tumorigenic abilities [61].

Monocytes are recruited into the tumour site by chemokines secreted by tumour and stroma cells including vascular endothelial growth factor (VEGF), semaphorin 3A (SEMA3A), CCL2 and C-X-C motif ligand (CXCL)12 [64,65]. In tumours, the signals that orchestrate macrophage functions can vary between different tumour types, or even between different parts of the same tumour resulting in diverse TAM phenotypes [66]. TAMs with a relatively M1-like skewed phenotype are found to be associated with the early phases of tumour development or with regressing tumours. Classically activated M1-like macrophages can kill tumour cells and they mediate tissue-destructive reactions by taking part in the early elimination phase of immuno-editing orchestrated by CD8^+^ cytotoxic T lymphocytes and interferons. Generally, TAM polarization toward an M2 phenotype seems to be a common feature for many cancers although their relative abundance depends on the tumour type. TAMs have properties correlated with angiogenesis, immunosuppression and promotion of cancer growth and metastasis [67,68]. For example, M2-like TAM are found in perivascular and hypoxic regions of mouse and human tumours [69]; angiopoietin receptor TIE-2^+^ monocyte/macrophages are important for angiogenesis [70] and they are associated with M2 skewed phenotype and tissue remodelling activity [71].

TAMs are involved in all the steps of cancer progression by supporting tumour cell invasion, angiogenesis, intravasation, motility, extravasation and metastasis formation [72]. Indeed, epidemiological evidence indicates that the presence of TAMs is often correlated with a poor disease prognosis in different forms of cancer, such as pancreatic, breast and lung [73].

## 5. Macrophages and Anti-Tumour Immunity

**Figure 2:**
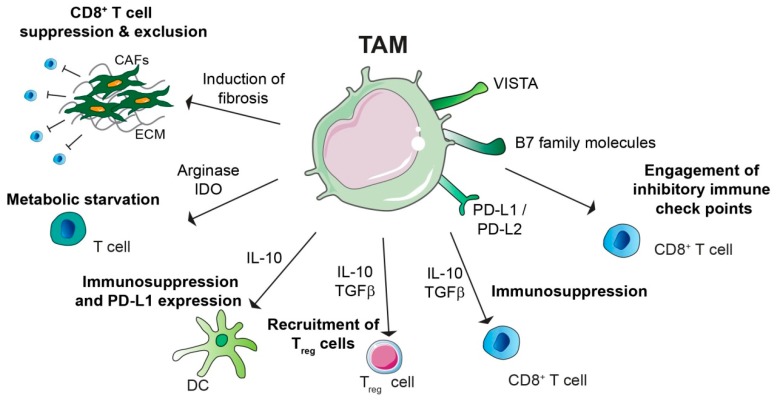
Immunosuppressive role of tumour-associated macrophages (TAMs). TAMs promote immunosuppression by different mechanisms: TAMs can express programmed cell death ligand 1 and ligand 2 (PD-L1 and PD-L2), which bind the T cell inhibitory receptor programmed cell death protein 1 (PD-1), thereby inducing T cell exhaustion. TAM expression of V-domain Ig suppressor of T cell activation (VISTA) and B7 family molecules, such as B7-H4, can have similar function. TAMs secrete immunosuppressive cytokines such as IL-10 and TGFβ, which can have direct suppressive functions on CD8^+^ T cells, or they can induce recruitment of immunosuppressive CD4^+^ T regulatory (T_reg_) cells. TAM secreted IL-10 can impair T cells effector functions indirectly either by inhibit dendritic cell (DC) anti-tumorigenic role or by inducing DC expression of PD-L1. Moreover, amino acid metabolism in TAM results in production of arginase and immunosuppressive metabolites via the indoleamine 2,3-dioxygenase (IDO) pathway, which is responsible of metabolic starvation in T cells. TAMs can also indirectly induce immunosuppression by promoting fibrosis. Indeed cancer associated fibroblast (CAF) secreted factors and/or extracellular matrix (ECM) stiffness are responsible for CD8^+^ T cell immunosuppression and/or exclusion from the tumour nest.

Macrophages, and in particular TAMs, critically affect anti-tumour immunity and response to immunotherapy (Figure 2).

TAMs can express PD-1 ligands PD-L1 and PD-L2, as well as CTLA-4 ligands CD80 and CD86. PD-L1 and PD-L2 are up-regulated in macrophages in response to different stimuli including cytokines and hypoxia [74,75]. A high level of PD-L1 expression in TAMs has been reported for different types of cancer such as hepatocellular carcinoma [76], glioblastoma [77], pancreatic cancer [78]. Interestingly, in a pre-clinical model of pancreatic cancer, macrophages are the main source of PD-L1 within the TME [79]. In melanoma and pancreatic cancer, CD68^+^ TAMs can exert their immunosuppressive functions by expressing VISTA [80]. TAM secreted TGFβ directly suppresses CD8^+^ T cell functions by transcriptional repression of genes encoding functional cytokines such as perforin, granzyme and cytotoxins [81]; TAM derived TGFβ [82] and IL-10 [83] can also act indirectly by stimulating the differentiation of T_reg_ cells. IL-10 stimulates PD-L1 expression on DCs [84] or it can suppress DCs anti-tumour function by inhibiting their secretion of IL-12 [85]. In addition, amino acid metabolism induced in M2-like macrophages or TAMs is responsible for arginase activity and/or production of immunosuppressive metabolites via the indoleamine 2,3-dioxygenase (IDO) pathway, resulting in metabolic starvation of T cells [86].

Macrophages also indirectly regulate CD8^+^ T infiltration and activity by inducing fibrosis. Fibrotic components, including cancer associated fibroblasts (CAFs) as well as the generation of hypoxic microenvironment due to extracellular matrix (ECM) stiffness and collagen deposition have been shown to be key mediators of T cell exclusion and immunosuppression [87]. The contribution of macrophages, in particular those with an alternative M2-like phenotype, to fibrosis has been extensively reported [88]. In particular, in pancreatic cancer the immunosuppressive role of fibrosis has raised large interest since a typical hallmark of this cancer is presence of an excessive fibrotic-inflammatory TME both at the primary and secondary liver metastatic site [7,42,89,90]. Indeed, it has been shown that liver Kupffer cells secrete TGFβ upon uptake of pancreatic cancer cell-derived exosomes, thereby triggering the generation of a fibrotic microenvironment responsible for establishing a pre-metastatic niche [90]. In contrast, the outgrowth of pancreatic cancer metastasis critically depends on the accumulation of bone marrow derived macrophages. These metastasis associated macrophages secrete high levels of granulin, a potent activator of hepatic stellate cells, thereby further fueling the generation of a fibrotic and inflammatory metastatic TME [7]. Macrophages are also important producers of matrix metalloproteinases (MMPs) and the administration of a CD40 agonist to pancreatic tumour bearing mice stimulated the systemic release of IFNγ and CCL2, thereby promoting the infiltration of MMP13 expressing monocytes/macrophages. In turn, macrophage-derived MMP13 reduced desmoplasia and enhanced chemotherapy delivery [91]. Moreover, targeting macrophages by blocking CSF-1/CSF-1R decreased fibrosis at the primary tumour site of the autochthonous *LSL-KrasG12D/^+^; LSL-Trp53R172H/^+^; Pdx-1-Cre* mice (KPC) mouse model of pancreatic cancer [79]. In regard to the hepatic metastatic site, metastasis associated macrophage (MAM) secrete high levels of the pro-fibrotic factor granulin, which is necessary for the generation of a fibrotic hepatic metastatic niche [7,92]. Subsequently, genetic depletion of granulin or the reduction of macrophage numbers by blocking CSF-1 reduced metastasis associated fibrosis and increased CD8^+^ T cell infiltration [42]. 

Considering the high immunomodulatory effects of macrophages, their targeting has become one of the most promising approaches to enhance anti-tumour immunity.

## 6. Targeting the Immunosuppressive Role of Macrophages

**Figure 3:**
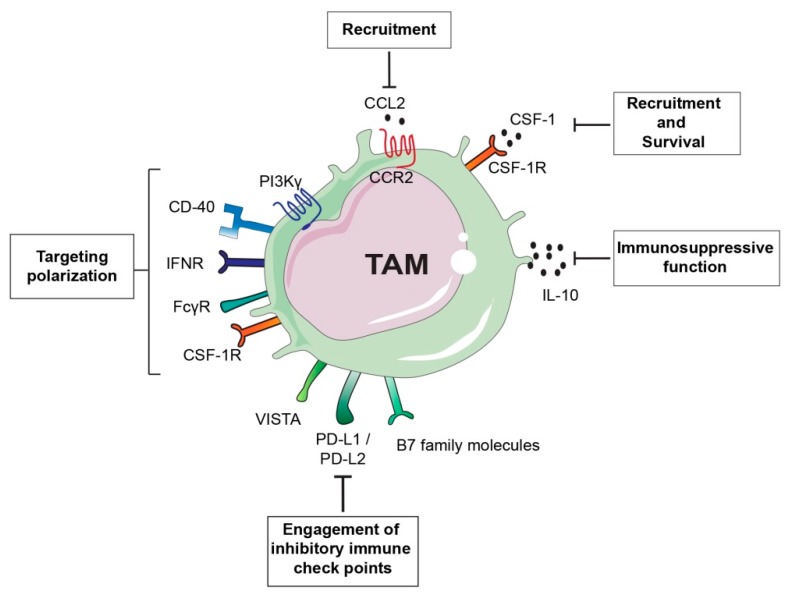
Tumour-associated macrophage (TAM)-targeted therapeutic approaches. TAM-centered approaches either aim at inhibiting TAM recruitment and survival or they focus on promoting TAM anti-tumorigenic activity. For example inhibition of the monocyte chemoattractant chemokine (C-C motif) ligand 2 (CCL2) or its receptor, as well as blockade of colony stimulation factor (CSF-1)/CSF-1 receptor (CSF-1R) axis can prevent TAM accumulation at the tumour site or TAM survival. CSF-1/CSF-1R blockade can also have the potential to switch TAM phenotype from a pro-tumorigenic M2-like to a pro-inflammatory M1-like. Similarly, approaches aimed at re-educating TAMs toward an anti-tumorigenic phenotype are successful as in the case of the use of inhibitors of gamma isoform of phosphoinositide 3-kinase (PI3Kγ) or Ig receptor gamma (FcRγ). Interferon gamma (IFNγ) administration or activation of the stimulatory receptor CD40 using anti-CD40 antibody agonists can induce re-education of pro-tumorigenic TAMs. Another alternative approach is the targeting the immunosuppressive function of TAMs by inhibiting IL-10 or blockade of immune checkpoint receptors engagement.

The signalling processes involved in TAM recruitment and/or activation are important targets for anti-cancer therapies (Figure 3).

CSF-1 is a monocyte attractant as well as the major growth and differentiation factor for monocyte-macrophage lineage. CSF-1 induces macrophage polarization toward an immunosuppressive and M2-like tumour promoting phenotype and it is abundantly expressed by several tumour types [93]. Therefore, CSF-1/CSF-1R axis has been extensively investigated and it is considered as an attractive target to interfere with TAM functions. A high level of CSF-1 or CSF-1R expression in the tumour or peri-tumoral tissue has been associated with poor patient survival in different malignancies such as lymphoma, breast cancer and hepatocellular carcinoma [94,95,96,97]. CSF-1R is a receptor tyrosine kinase and a number of small molecules and antibody antagonists have been developed and tested in pre-clinical models. For example, mice treatment with the humanized mAb Emactuzumab, which binds CSF-1R, prevents receptor dimerization thereby abrogating CSF-1 receptor binding and activation of downstream signalling. In this pre-clinical study, CSF-1R inhibition reduced TAM and circulating monocyte numbers and increased the CD8^+^: CD4^+^ T cell ratio compared with mice treated with control antibody [98]. The use of combinational therapies has been developed to potentiate the effect of CSF-1/CSF-1R inhibitors. For example, radiotherapy has been demonstrated to increase CSF-1 expression and myeloid cell infiltration in preclinical mouse xenograft models of human glioblastomas and combinational treatment of radiotherapy with CSF-1R small molecule inhibitors has shown to potentiate radiotherapy efficacy [99]. Another small molecule inhibitor, BLZ945, has shown to decrease glioma progression and improved survival in preclinical models. Interestingly, CSF-1R blockade in this model did not induce decrease in TAM numbers, but induced phenotypic changes in macrophage populations from an M2-like pro-tumoral to M1-like anti-tumoral type instead [100]. Blockade of CSF-1/CSF-1R axis in a pre-clinical mouse model of pancreatic cancer metastasis impaired macrophage recruitment and induced a phenotypic switch of remaining MAMs toward a pro-inflammatory, M1-like phenotype [42]. Another study revealed that pharmacological blockade of CSF-1/CSF-1R targeted specifically breast cancer CD11b^+^ Ly6G^neg^ Ly6C^low^ F4/80^+^ TAMs and induced increase in CD8^+^ lymphocyte infiltration [101]. The same group also identified macrophages as a primary source of IL-10 and that inhibition of IL-10 receptor induced reduction of the breast cancer tumour burden if combined with chemotherapy, with an equivalent effect caused by blockade of CSF-1R. Also in this case, tumour reduction was associated with increased CD8^+^ T cell mediated anti-tumour activity. Mechanistically, macrophage derived IL-10 suppressed IL-12 production by intra-tumoral DCs, which in turn limited CD8^+^ T cell cytotoxic activity [85]. Similar results have been obtained in the case of pancreatic cancer. Blockade of CSF-1R or CCR2 resulted in altered infiltration of myeloid cells by affecting CD11b^+^ Ly6G^neg^ Ly6C^low^ MHCII^high^ F480^+^ macrophages and CD11b^+^ Ly6G^neg^ Ly6C^high^ MHCII^+^ F480^+^ monocytes, respectively. CSF-1R or CCR2 inhibition in combination with chemotherapy resulted in restored CD8^+^ T cells’ anti-tumoral activity [102]. The enhanced anti-tumoral effect of CD8^+^ T cells as consequence of pharmacological inhibition of CSF-1/CSF-1R axis has also been exploited for combinational therapy with ICBs, especially for tumours resistant to single-agent therapy. In pancreatic cancer, CD11b^+^ Ly6G^neg^ Ly6C^low^ F480^+^ CD206^high^ M2-like polarized macrophages have been observed to be more sensitive to CSF-1/CSF-1R blockade. In this model, CSF-1/CSF-1R targeting resulted in increased expression of immune checkpoint molecule on tumour cells, including PD-L1 and CTLA-4, thus resulting in only modest increase of CD8^+^ T cell cytotoxicity. Combination of anti- PD1 or anti- CTLA-4 checkpoint immunotherapy with CSF-1/CSF-1R blockade improved anti-tumour immunity and led to the regression of even established primary pancreatic tumours [103]. However, targeting CSF-1/CSF1-R axis could also lead to compensatory effects, which translate to enhanced tumour progression. For instance, using different mouse tumour models, Kumar and colleagues demonstrated that CSF1-R is not only expressed on macrophages, but also on CAFs. Interestingly, in this study the inhibition of CSF-1R increased the secretion of CAF-derived chemokines including CXCL1, thereby provoking the recruitment of immunosuppressive Ly6G^+^ granulocytes/neutrophils. Inhibition of CXCR2, which is the receptor for most of the up-regulated CAF-secreted cytokines in response to CSF-1R inhibition, alongside with CSF-1R resulted in significant delay in cancer progression [104].

Combination of ICBs with myeloid cells targeting agents has recently been tested in preclinical pancreatic cancer models. For example, pharmacological selective targeting of the gamma isoform of phosphoinositide 3-kinase (PI3K**γ**), highly expressed in myeloid cells [105], reshaped TME, induced T cell mediated cytotoxic activity, and improved ICB therapies [106,107].

Other TAM-centred approaches aim at re-education of macrophages rather than simply targeting them for depletion or destruction. IFN**γ** is a classical inducer of M1-like polarization and its administration in women with ovarian cancer resulted in increased activation of tumour cytotoxicity and clinical response [108]. Another approach to targeting TAMs has been provided by administration of agonistic anti-CD40 antibody in mice with pancreatic cancer. CD40 agonist treatment resulted in re-education of M2-like macrophages toward an M1-like type with increased antigen presentation capabilities which led to re-establishment of pro-inflammatory microenvironment and a reduction in tumour mass [109]. Translation of the results obtained into clinical trials using a fully humanized antibody CD40 agonist in combination with chemotherapy in patients with advanced stage pancreatic cancer achieved partial response and improved patient survival [110]. Interestingly, a combination of anti-CD40 agonist and inhibition of CSF-1R induced macrophage pro-inflammatory phenotype prior CSF-1R mediated elimination, which was sufficient to reinvigorate CD8^+^ T cell response in transplanted tumours that were either resistant or sensitive to ICBs [111]. B cells can also affect macrophage activation and functions. The crosstalk between B cells and the Ig receptor gamma (FcRγ^+^) expressed on TAMs stimulates a Bruton tyrosine kinase (BTK)-PI3Kγ signalling cascade that favours an M2-like macrophage phenotype. Administration of a PI3Kγ inhibitor or a BTK inhibitor reverted macrophages towards an M1-like phenotype and promoted CD8^+^ T cell cytotoxicity resulting in a decrease in tumour growth [112].

Given the importance of TAMs as regulators of tumour immunity, therapeutic strategies aimed at both depleting TAMs and at targeting their pro-tumorigenic abilities are now under early phase clinical evaluation in combination with ICB (Table 1).

## 7. Conclusions

The use of ICBs, in particular the targeting of the PD-1/PD-L1 pathway, is considered one of the most promising anti-cancer treatments and impressive therapeutic benefits have been reported for its use in different cancer types. However resistance to ICB is common [113]. Indeed, priming and sustaining an anti-tumour immune response in solid tumours still remains challenging due to obstacles posed by solid tumours, particularly T cell exclusion and the presence of a myriad of immunosuppressive factors within the TME. Increasing evidence suggests that TME-targeted therapies restoring T cell infiltration and activation are inevitable for subsequent tumour response to ICBs. Macrophages are key regulators of tissue development and homeostasis but tumours have acquired the ability to educate them for their own benefit. As discussed in this review, TAMs play a crucial role in suppressing T cell recruitment and function, thereby favouring tumour immune escape. Nowadays TAM-centred therapeutic strategies are generally focused on inhibiting recruitment and/or survival of macrophages at the tumour site, alternatively other strategies are focused at enhancing macrophage anti-tumour activities. Therapeutic approaches aimed at TAM reprogramming rather than targeting for depletion seems to be promising considering that macrophages with an M2-like phenotype are mainly associated with immunosuppressive and tumour supportive functions. This is also important in the light of evidence that TAM depletion can lead to compensatory effects mediated by Ly6G^+^ neutrophils or T_reg_ cells [42,114]. Therefore, a promising attempt to overcome resistance to ICB is the stimulation of pro-inflammatory and anti-tumorigenic functions of TAMs to prime cytotoxic T cell responses.

Moreover, there is emerging evidence that the TME markedly varies between organs, cancer type, and primary and secondary sites. Thus, the identification and molecular characterization of immunosuppressive mechanisms within the distinct TMEs will be critical for the development of effective TME-targeted therapies.

## Figures and Tables

**Table 1:** Clinical trials of TAM targeting agents in combination with ICBs.

***TAM Targeting Agent***	***Immune Checkpoint Blocker***	***Tumour Type***	***Phase***	***ClinicalTrial.Gov Reference***
**CSF-1/CSF-1R Antagonists**				
PLX3397	Pembrolizumab (anti-PD-L1)	Advanced solid tumours	Phase II (completed)	NCT02452424
Pexidartinib	Durvalumab (anti-PD-L1)	Metastatic/advanced pancreatic or colorectal cancers	Phase I (ongoing)	NCT02777710
BLZ945	PDR001 (anti-PD-1)	Advanced solid tumours	Phase I/II (ongoing)	NCT02829723
Emactuzumab	Atezolizumab (anti-PD-L1)	Advanced solid tumours	Phase I (ongoing)	NCT02323191
AMG820	Pembrolizumab (anti-PD-L1)	Pancreatic cancer, colorectal cancer, non-small cell lung cancer	Phase I/II (ongoing)	NCT02713529
**CCR2/CCR5 Antagonist**				
BMS-813160	Nivolumab (anti-PD-1)	Metastatic pancreatic or colorectal cancers	Phase I/II (ongoing)	NCT03184870
**PI3K δ/γ Antagonists**				
Tenalisib	Pembrolizumab (anti-PD-L1)	Classical Hodgkin Lymphoma	Phase I/II (completed)	NCT03471351
IPI-549	Nivolumab (anti-PD-1)	Advanced solid tumours	Phase I (ongoing)	NCT02637531
**BTK Antagonist**				
Ibrutinib	Durvalumab (anti-PD-L1)	Relapsed or refractory solid tumours	Phase I/II (terminated)	NCT02403271
**CD40 Agonists**				
APX005M	Nivolumab (anti-PD-1)	Non-small cell lung cancer or metastatic melanoma	Phase I/II (ongoing)	NCT03123783
Selicrelumab	Atezolizumab (anti PD-L1)	Locally advanced and/or metastatic solid tumours	Phase I (ongoing)	NCT02304393

Clinical trials of tumour-associated macrophage (TAM) targeting agents in combination with ICBs. Data were obtained from https://clinicaltrials.gov. CSF, colony stimulating factor; CSF-1R, colony stimulating factor receptor; PD-1, programmed cell-death protein 1; PD-1, programmed cell-death 1 ligand 1; CCR2, C-C motif chemokine receptor 2; CCR5, C-C motif chemokine receptor 5; PI3K, phosphoinositide 3-kinase; BTK, Bruton tyrosine kinase; CD40, cluster of differentiation 40.

## References

[1] Hanahan D., Weinberg R.A. (2011). Hallmarks of cancer: The next generation. Cell.

[2] Joyce J.A., Pollard J.W. (2009). Microenvironmental regulation of metastasis. Nat. Rev. Cancer.

[3] Ribas A., Wolchok J.D. (2018). Cancer immunotherapy using checkpoint blockade. Science (80-)..

[4] Chen D.S., Mellman I. (2013). Oncology meets immunology: The cancer-immunity cycle. Immunity.

[5] Hanahan D., Coussens L.M.M. (2012). Accessories to the crime: Functions of cells recruited to the tumor microenvironment. Cancer Cell.

[6] Ireland L., Santos A., Ahmed M.S., Rainer C., Nielsen S.R., Quaranta V., Weyer-Czernilofsky U., Engle D.D., Perez-Mancera P.A., Coupland S.E. (2016). Chemoresistance in Pancreatic Cancer Is Driven by Stroma-Derived Insulin-Like Growth Factors. Cancer Res..

[7] Nielsen S.R., Quaranta V., Linford A., Emeagi P., Rainer C., Santos A., Ireland L., Sakai T., Sakai K., Kim Y.-S. (2016). Macrophage-secreted granulin supports pancreatic cancer metastasis by inducing liver fibrosis. Nat. Cell Biol..

[8] Shankaran V., Ikeda H., Bruce A.T., White J.M., Swanson P.E., Old L.J., Schreiber R.D. (2001). IFNγ and lymphocytes prevent primary tumour development and shape tumour immunogenicity. Nature.

[9] Goodnow C.C., Sprent J., de St Groth B.F., Vinuesa C.G. (2005). Cellular and genetic mechanisms of self tolerance and autoimmunity. Nature.

[10] Walunas T.L., Lenschow D.J., Bakker C.Y., Linsley P.S., Freeman G.J., Green J.M., Thompson C.B., Bluestone J.A. (1994). CTLA-4 can function as a negative regulator of T cell activation. Immunity.

[11] Leach D.R., Krummel M.F., Allison J.P. (1996). Enhancement of antitumor immunity by CTLA-4 blockade. Science.

[12] van Elsas A., Hurwitz A.A., Allison J.P. (1999). Combination immunotherapy of B16 melanoma using anti-cytotoxic T lymphocyte-associated antigen 4 (CTLA-4) and granulocyte/macrophage colony-stimulating factor (GM-CSF)-producing vaccines induces rejection of subcutaneous and metastatic tumors accompanied by autoimmune depigmentation. J. Exp. Med..

[13] Robert C., Thomas L., Bondarenko I., O’Day S., Weber J., Garbe C., Lebbe C., Baurain J.-F., Testori A., Grob J.-J. (2011). Ipilimumab plus Dacarbazine for Previously Untreated Metastatic Melanoma. N. Engl. J. Med..

[14] Hodi F.S., O’Day S.J., McDermott D.F., Weber R.W., Sosman J.A., Haanen J.B., Gonzalez R., Robert C., Schadendorf D., Hassel J.C. (2010). Improved Survival with Ipilimumab in Patients with Metastatic Melanoma. N. Engl. J. Med..

[15] Yang J.C., Hughes M., Kammula U., Royal R., Sherry R.M., Topalian S.L., Suri K.B., Levy C., Allen T., Mavroukakis S. (2007). Ipilimumab (Anti-CTLA4 Antibody) Causes Regression of Metastatic Renal Cell Cancer Associated With Enteritis and Hypophysitis. J. Immunother..

[16] Kwon E.D., Drake C.G., Scher H.I., Fizazi K., Bossi A., van den Eertwegh A.J.M., Krainer M., Houede N., Santos R., Mahammedi H. (2014). Ipilimumab versus placebo after radiotherapy in patients with metastatic castration-resistant prostate cancer that had progressed after docetaxel chemotherapy (CA184-043): A multicentre, randomised, double-blind, phase 3 trial. Lancet Oncol..

[17] Carthon B.C., Wolchok J.D., Yuan J., Kamat A., Ng Tang D.S., Sun J., Ku G., Troncoso P., Logothetis C.J., Allison J.P. (2010). Preoperative CTLA-4 Blockade: Tolerability and Immune Monitoring in the Setting of a Presurgical Clinical Trial. Clin. Cancer Res..

[18] Hurwitz A.A., Yu T.F., Leach D.R., Allison J.P., Haluska F.G., Kruse A., MacRae S., Nelson M., Canning C., Lowy I. (1998). CTLA-4 blockade synergizes with tumor-derived granulocyte-macrophage colony-stimulating factor for treatment of an experimental mammary carcinoma. Proc. Natl. Acad. Sci. USA.

[19] Freeman G.J., Long A.J., Iwai Y., Bourque K., Chernova T., Nishimura H., Fitz L.J., Malenkovich N., Okazaki T., Byrne M.C. (2000). Engagement of the PD-1 immunoinhibitory receptor by a novel B7 family member leads to negative regulation of lymphocyte activation. J. Exp. Med..

[20] Latchman Y., Wood C.R., Chernova T., Chaudhary D., Borde M., Chernova I., Iwai Y., Long A.J., Brown J.A., Nunes R. (2001). PD-L2 is a second ligand for PD-1 and inhibits T cell activation. Nat. Immunol..

[21] Wei S.C., Levine J.H., Cogdill A.P., Zhao Y., Anang N.-A.A.S., Andrews M.C., Sharma P., Wang J., Wargo J.A., Pe’er D. (2017). Distinct Cellular Mechanisms Underlie Anti-CTLA-4 and Anti-PD-1 Checkpoint Blockade. Cell.

[22] Lau J., Cheung J., Navarro A., Lianoglou S., Haley B., Totpal K., Sanders L., Koeppen H., Caplazi P., McBride J. (2017). Tumour and host cell PD-L1 is required to mediate suppression of anti-tumour immunity in mice. Nat. Commun..

[23] Garcia-Diaz A., Shin D.S., Homet B., Damoiseaux R., Lo R.S., Ribas A. (2017). Interferon Receptor Signaling Pathways Regulating PD-L1 and PD-L2 Expression. CellReports.

[24] Blackburn S.D., Shin H., Haining W.N., Zou T., Workman C.J., Polley A., Betts M.R., Freeman G.J., Vignali D.A.A., Wherry E.J. (2009). Coregulation of CD8^+^ T cell exhaustion by multiple inhibitory receptors during chronic viral infection. Nat. Immunol..

[25] Wherry E.J., Ha S.-J., Kaech S.M., Haining W.N., Sarkar S., Kalia V., Subramaniam S., Blattman J.N., Barber D.L., Ahmed R. (2007). Molecular Signature of CD8^+^ T Cell Exhaustion during Chronic Viral Infection. Immunity.

[26] Robert C., Schachter J., Long G.V., Arance A., Grob J.J., Mortier L., Daud A., Carlino M.S., McNeil C., Lotem M. (2015). Pembrolizumab versus Ipilimumab in Advanced Melanoma. N. Engl. J. Med..

[27] Eroglu Z., Zaretsky J.M., Hu-Lieskovan S., Kim D.W., Algazi A., Johnson D.B., Liniker E., Ben Kong B., Munhoz R., Rapisuwon S. (2018). High response rate to PD-1 blockade in desmoplastic melanomas. Nature.

[28] Garon E.B., Rizvi N.A., Hui R., Leighl N., Balmanoukian A.S., Eder J.P., Patnaik A., Aggarwal C., Gubens M., Horn L. (2015). Pembrolizumab for the Treatment of Non–Small-Cell Lung Cancer. N. Engl. J. Med..

[29] Ferris R.L., Blumenschein G., Fayette J., Guigay J., Colevas A.D., Licitra L., Harrington K., Kasper S., Vokes E.E., Even C. (2016). Nivolumab for Recurrent Squamous-Cell Carcinoma of the Head and Neck. N. Engl. J. Med..

[30] Ansell S.M., Lesokhin A.M., Borrello I., Halwani A., Scott E.C., Gutierrez M., Schuster S.J., Millenson M.M., Cattry D., Freeman G.J. (2015). PD-1 Blockade with Nivolumab in Relapsed or Refractory Hodgkin’s Lymphoma. N. Engl. J. Med..

[31] Rosenberg J.E., Hoffman-Censits J., Powles T., van der Heijden M.S., Balar A.V., Necchi A., Dawson N., O’Donnell P.H., Balmanoukian A., Loriot Y. (2016). Atezolizumab in patients with locally advanced and metastatic urothelial carcinoma who have progressed following treatment with platinum-based chemotherapy: A single-arm, multicentre, phase 2 trial. Lancet.

[32] Bellmunt J., de Wit R., Vaughn D.J., Fradet Y., Lee J.-L., Fong L., Vogelzang N.J., Climent M.A., Petrylak D.P., Choueiri T.K. (2017). Pembrolizumab as Second-Line Therapy for Advanced Urothelial Carcinoma. N. Engl. J. Med..

[33] Curran M.A., Montalvo W., Yagita H., Allison J.P. (2010). PD-1 and CTLA-4 combination blockade expands infiltrating T cells and reduces regulatory T and myeloid cells within B16 melanoma tumors. Proc. Natl. Acad. Sci. USA.

[34] Wolchok J.D., Kluger H., Callahan M.K., Postow M.A., Rizvi N.A., Lesokhin A.M., Segal N.H., Ariyan C.E., Gordon R.-A., Reed K. (2013). Nivolumab plus Ipilimumab in Advanced Melanoma. N. Engl. J. Med..

[35] Brahmer J.R., Tykodi S.S., Chow L.Q.M., Hwu W.-J., Topalian S.L., Hwu P., Drake C.G., Camacho L.H., Kauh J., Odunsi K. (2012). Safety and Activity of Anti–PD-L1 Antibody in Patients with Advanced Cancer. N. Engl. J. Med..

[36] Gubin M.M., Zhang X., Schuster H., Caron E., Ward J.P., Noguchi T., Ivanova Y., Hundal J., Arthur C.D., Krebber W.-J. (2014). Checkpoint blockade cancer immunotherapy targets tumour-specific mutant antigens. Nature.

[37] Balachandran V.P., Luksza M., Zhao J.N., Makarov V., Moral J.A., Remark R., Herbst B., Askan G., Bhanot U., Senbabaoglu Y. (2017). Identification of unique neoantigen qualities in long-term survivors of pancreatic cancer. Nature.

[38] Havel J.J., Chowell D., Chan T.A. (2019). The evolving landscape of biomarkers for checkpoint inhibitor immunotherapy. Nat. Rev. Cancer.

[39] Joyce J.A., Fearon D.T. (2015). T cell exclusion, immune privilege, and the tumor microenvironment. Science.

[40] Zhang L., Conejo-Garcia J.R., Katsaros D., Gimotty P.A., Massobrio M., Regnani G., Makrigiannakis A., Gray H., Schlienger K., Liebman M.N. (2003). Intratumoral T Cells, Recurrence, and Survival in Epithelial Ovarian Cancer. N. Engl. J. Med..

[41] Sato E., Olson S.H., Ahn J., Bundy B., Nishikawa H., Qian F., Jungbluth A.A., Frosina D., Gnjatic S., Ambrosone C. (2005). Intraepithelial CD8^+^ tumor-infiltrating lymphocytes and a high CD8^+^/regulatory T cell ratio are associated with favorable prognosis in ovarian cancer. Proc. Natl. Acad. Sci. USA.

[42] Quaranta V., Rainer C., Nielsen S.R., Raymant M.L., Ahmed M.S., Engle D.D., Taylor A., Murray T., Campbell F., Palmer D.H. (2018). Macrophage-Derived Granulin Drives Resistance to Immune Checkpoint Inhibition in Metastatic Pancreatic Cancer. Cancer Res..

[43] Buckanovich R.J., Facciabene A., Kim S., Benencia F., Sasaroli D., Balint K., Katsaros D., O’Brien-Jenkins A., Gimotty P.A., Coukos G. (2008). Endothelin B receptor mediates the endothelial barrier to T cell homing to tumors and disables immune therapy. Nat. Med..

[44] Mariathasan S., Turley S.J., Nickles D., Castiglioni A., Yuen K., Wang Y., Kadel E.E., Koeppen H., Astarita J.L., Cubas R. (2018). TGFβ attenuates tumour response to PD-L1 blockade by contributing to exclusion of T cells. Nature.

[45] Erwig L.-P., Henson P.M. (2008). Clearance of apoptotic cells by phagocytes. Cell Death Differ..

[46] Biswas S.K., Allavena P., Mantovani A. (2013). Tumor-associated macrophages: Functional diversity, clinical significance, and open questions. Semin. Immunopathol..

[47] Murray P.J., Wynn T.A. (2011). Protective and pathogenic functions of macrophage subsets. Nat. Rev. Immunol..

[48] Mass E., Ballesteros I., Farlik M., Halbritter F., Günther P., Crozet L., Jacome-Galarza C.E., Händler K., Klughammer J., Kobayashi Y. (2016). Specification of tissue-resident macrophages during organogenesis. Science.

[49] Ginhoux F., Merad M. (2010). Ontogeny and homeostasis of Langerhans cells. Immunol. Cell Biol..

[50] Ginhoux F., Schultze J.L., Murray P.J., Ochando J., Biswas S.K. (2016). New insights into the multidimensional concept of macrophage ontogeny, activation and function. Nat. Immunol..

[51] Schulz C., Perdiguero E.G., Chorro L., Szabo-Rogers H., Cagnard N., Kierdorf K., Prinz M., Wu B., Jacobsen S.E.W., Pollard J.W. (2012). A Lineage of Myeloid Cells Independent of Myb and Hematopoietic Stem Cells. Science (80-)..

[52] Sheng J., Ruedl C., Karjalainen K. (2015). Most Tissue-Resident Macrophages Except Microglia Are Derived from Fetal Hematopoietic Stem Cells. Immunity.

[53] DeNardo D.G., Ruffell B. (2019). Macrophages as regulators of tumour immunity and immunotherapy. Nat. Rev. Immunol..

[54] Geissmann F., Manz M.G., Jung S., Sieweke M.H., Merad M., Ley K. (2010). Development of Monocytes, Macrophages, and Dendritic Cells. Science..

[55] Hanna R.N., Cekic C., Sag D., Tacke R., Thomas G.D., Nowyhed H., Herrley E., Rasquinha N., McArdle S., Wu R. (2015). Patrolling monocytes control tumor metastasis to the lung. Science (80-. )..

[56] Biswas S.K., Mantovani A. (2010). Macrophage plasticity and interaction with lymphocyte subsets: Cancer as a paradigm. Nat. Immunol..

[57] Franklin R.A., Liao W., Sarkar A., Kim M.V., Bivona M.R., Liu K., Pamer E.G., Li M.O. (2014). The cellular and molecular origin of tumor-associated macrophages. Science (80-. )..

[58] Youn J.-I., Kumar V., Collazo M., Nefedova Y., Condamine T., Cheng P., Villagra A., Antonia S., McCaffrey J.C., Fishman M. (2013). Epigenetic silencing of retinoblastoma gene regulates pathologic differentiation of myeloid cells in cancer. Nat. Immunol..

[59] Gabrilovich D.I., Ostrand-Rosenberg S., Bronte V. (2012). Coordinated regulation of myeloid cells by tumours. Nat. Rev. Immunol..

[60] Kumar V., Cheng P., Condamine T., Mony S., Languino L.R., McCaffrey J.C., Hockstein N., Guarino M., Masters G., Penman E. (2016). CD45 Phosphatase Inhibits STAT3 Transcription Factor Activity in Myeloid Cells and Promotes Tumor-Associated Macrophage Differentiation. Immunity.

[61] Zhu Y., Herndon J.M., Sojka D.K., Kim K.-W., Knolhoff B.L., Zuo C., Cullinan D.R., Luo J., Bearden A.R., Lavine K.J. (2017). Tissue-Resident Macrophages in Pancreatic Ductal Adenocarcinoma Originate from Embryonic Hematopoiesis and Promote Tumor Progression. Immunity.

[62] Loyher P.-L., Hamon P., Laviron M., Meghraoui-Kheddar A., Goncalves E., Deng Z., Torstensson S., Bercovici N., Baudesson de Chanville C., Combadière B. (2018). Macrophages of distinct origins contribute to tumor development in the lung. J. Exp. Med.

[63] Bowman R.L., Klemm F., Akkari L., Pyonteck S.M., Sevenich L., Quail D.F., Dhara S., Simpson K., Gardner E.E., Iacobuzio-Donahue C.A. (2016). Macrophage Ontogeny Underlies Differences in Tumor-Specific Education in Brain Malignancies. Cell Rep..

[64] Qian B.-Z., Li J., Zhang H., Kitamura T., Zhang J., Campion L.R., Kaiser E.A., Snyder L.A., Pollard J.W. (2011). CCL2 recruits inflammatory monocytes to facilitate breast-tumour metastasis. Nature.

[65] Casazza A., Laoui D., Wenes M., Rizzolio S., Bassani N., Mambretti M., Deschoemaeker S., Van Ginderachter J.A., Tamagnone L., Mazzone M. (2013). Impeding Macrophage Entry into Hypoxic Tumor Areas by Sema3A/Nrp1 Signaling Blockade Inhibits Angiogenesis and Restores Antitumor Immunity. Cancer Cell.

[66] Noy R., Pollard J.W. (2014). Tumor-Associated Macrophages: From Mechanisms to Therapy. Immunity.

[67] Pucci F., Venneri M.A., Biziato D., Nonis A., Moi D., Sica A., Di Serio C., Naldini L., De Palma M. (2009). A distinguishing gene signature shared by tumor-infiltrating Tie2-expressing monocytes, blood “resident” monocytes, and embryonic macrophages suggests common functions and developmental relationships. Blood.

[68] Movahedi K., Laoui D., Gysemans C., Baeten M., Stange G., Van den Bossche J., Mack M., Pipeleers D., In’t Veld P., De Baetselier P. (2010). Different Tumor Microenvironments Contain Functionally Distinct Subsets of Macrophages Derived from Ly6C(high) Monocytes. Cancer Res..

[69] Mazzieri R., Pucci F., Moi D., Zonari E., Ranghetti A., Berti A., Politi L.S., Gentner B., Brown J.L., Naldini L. (2011). Targeting the ANG2/TIE2 Axis Inhibits Tumor Growth and Metastasis by Impairing Angiogenesis and Disabling Rebounds of Proangiogenic Myeloid Cells. Cancer Cell.

[70] De Palma M., Murdoch C., Venneri M.A., Naldini L., Lewis C.E. (2007). Tie2-expressing monocytes: Regulation of tumor angiogenesis and therapeutic implications. Trends Immunol..

[71] Squadrito M.L., Pucci F., Magri L., Moi D., Gilfillan G.D., Ranghetti A., Casazza A., Mazzone M., Lyle R., Naldini L. (2012). miR-511-3p Modulates Genetic Programs of Tumor-Associated Macrophages. Cell Rep..

[72] Mantovani A., Marchesi F., Malesci A., Laghi L., Allavena P. (2017). Tumour-associated macrophages as treatment targets in oncology. Nat. Rev. Clin. Oncol..

[73] Bingle L., Brown N.J., Lewis C.E. (2002). The role of tumour-associated macrophages in tumour progression: Implications for new anticancer therapies. J. Pathol..

[74] Noman M.Z., Desantis G., Janji B., Hasmim M., Karray S., Dessen P., Bronte V., Chouaib S. (2014). PD-L1 is a novel direct target of HIF-1α, and its blockade under hypoxia enhanced MDSC-mediated T cell activation. J. Exp. Med..

[75] Loke P., Allison J.P. (2003). PD-L1 and PD-L2 are differentially regulated by Th1 and Th2 cells. Proc. Natl. Acad. Sci. USA.

[76] Kuang D.-M., Zhao Q., Peng C., Xu J., Zhang J.-P., Wu C., Zheng L. (2009). Activated monocytes in peritumoral stroma of hepatocellular carcinoma foster immune privilege and disease progression through PD-L1. J. Exp. Med..

[77] Bloch O., Crane C.A., Kaur R., Safaee M., Rutkowski M.J., Parsa A.T. (2013). Gliomas Promote Immunosuppression through Induction of B7-H1 Expression in Tumor-Associated Macrophages. Clin. Cancer Res..

[78] Winograd R., Byrne K.T., Evans R.A., Odorizzi P.M., Meyer A.R.L., Bajor D.L., Clendenin C., Stanger B.Z., Furth E.E., Wherry E.J. (2015). Induction of T-cell Immunity Overcomes Complete Resistance to PD-1 and CTLA-4 Blockade and Improves Survival in Pancreatic Carcinoma. Cancer Immunol. Res..

[79] Candido J.B., Morton J.P., Bailey P., Campbell A.D., Karim S.A., Jamieson T., Lapienyte L., Gopinathan A., Clark W., McGhee E.J. (2018). CSF1R^+^Macrophages Sustain Pancreatic Tumor Growth through T Cell Suppression and Maintenance of Key Gene Programs that Define the Squamous Subtype. Cell Rep..

[80] Blando J., Sharma A., Higa M.G., Zhao H., Vence L., Yadav S.S., Kim J., Sepulveda A.M., Sharp M., Maitra A. (2019). Comparison of immune infiltrates in melanoma and pancreatic cancer highlights VISTA as a potential target in pancreatic cancer. Proc. Natl. Acad. Sci. USA.

[81] Thomas D.A., Massagué J. (2005). TGF-β directly targets cytotoxic T cell functions during tumor evasion of immune surveillance. Cancer Cell.

[82] Chen W., Jin W., Hardegen N., Lei K., Li L., Marinos N., McGrady G., Wahl S.M. (2003). Conversion of Peripheral CD4 ^+^ CD25 ^−^ Naive T Cells to CD4 ^+^ CD25 ^+^ Regulatory T Cells by TGF-β Induction of Transcription Factor *Foxp3*. J. Exp. Med..

[83] Roncarolo M.G., Bacchetta R., Bordignon C., Narula S., Levings M.K. (2001). Type 1 T regulatory cells. Immunol. Rev..

[84] Kim Y.-J., Park S.-J., Broxmeyer H.E. (2011). Phagocytosis, a Potential Mechanism for Myeloid-Derived Suppressor Cell Regulation of CD8^+^ T Cell Function Mediated through Programmed Cell Death-1 and Programmed Cell Death-1 Ligand Interaction. J. Immunol..

[85] Ruffell B., Chang-strachan D., Chan V., Rosenbusch A., Ho C.M.T., Pryer N., Daniel D., Hwang E.S., Rugo H.S., Coussens L.M. (2014). Article Responses to Chemotherapy by Suppressing IL-12 Expression in Intratumoral Dendritic Cells. Cancer Cell.

[86] Murray P.J.J., Allen J.E.E., Biswas S.K.K., Fisher E.A.A., Gilroy D.W.W., Goerdt S., Gordon S., Hamilton J.A.A., Ivashkiv L.B.B., Lawrence T. (2014). Macrophage Activation and Polarization: Nomenclature and Experimental Guidelines. Immunity.

[87] Jiang H., Hegde S., DeNardo D.G. (2017). Tumor-associated fibrosis as a regulator of tumor immunity and response to immunotherapy. Cancer Immunol. Immunother..

[88] Wynn T., Barron L. (2010). Macrophages: Master Regulators of Inflammation and Fibrosis. Semin. Liver Dis..

[89] Longo D.L., Ryan D.P., Hong T.S., Bardeesy N. (2014). Pancreatic Adenocarcinoma. N. Engl. J. Med..

[90] Costa-Silva B., Aiello N.M., Ocean A.J., Singh S., Zhang H., Thakur B.K., Becker A., Hoshino A., Mark M.T., Molina H. (2015). Pancreatic cancer exosomes initiate pre-metastatic niche formation in the liver. Nat. Cell Biol..

[91] Long K.B., Gladney W.L., Tooker G.M., Graham K., Fraietta J.A., Beatty G.L. (2016). IFN and CCL2 Cooperate to Redirect Tumor-Infiltrating Monocytes to Degrade Fibrosis and Enhance Chemotherapy Efficacy in Pancreatic Carcinoma. Cancer Discov..

[92] He Z., Ong C.H.P., Halper J., Bateman A. (2003). Progranulin is a mediator of the wound response. Nat. Med..

[93] Lenzo J.C., Turner A.L., Cook A.D., Vlahos R., Anderson G.P., Reynolds E.C., Hamilton J.A. (2012). Control of macrophage lineage populations by CSF-1 receptor and GM-CSF in homeostasis and inflammation. Immunol. Cell Biol..

[94] Koh Y.W., Park C., Yoon D.H., Suh C., Huh J. (2014). CSF-1R Expression in Tumor-Associated Macrophages Is Associated With Worse Prognosis in Classical Hodgkin Lymphoma. Am. J. Clin. Pathol..

[95] Zhu X.-D., Zhang J.-B., Zhuang P.-Y., Zhu H.-G., Zhang W., Xiong Y.-Q., Wu W.-Z., Wang L., Tang Z.-Y., Sun H.-C. (2008). High expression of macrophage colony-stimulating factor in peritumoral liver tissue is associated with poor survival after curative resection of hepatocellular carcinoma. J. Clin. Oncol..

[96] Goswami S., Sahai E., Wyckoff J.B., Cammer M., Cox D., Pixley F.J., Stanley E.R., Segall J.E., Condeelis J.S. (2005). Macrophages Promote the Invasion of Breast Carcinoma Cells via a Colony-Stimulating Factor-1/Epidermal Growth Factor Paracrine Loop. Cancer Res..

[97] Lin E.Y., Nguyen A.V., Russell R.G., Pollard J.W. (2001). Colony-stimulating factor 1 promotes progression of mammary tumors to malignancy. J. Exp. Med..

[98] Wartha K., Runza V., Ries C.H.H., Cannarile M.A.A., Hoves S., Rey-giraud F., Pradel L.P.P., Feuerhake F., Klaman I., Jones T. (2014). Article Targeting Tumor-Associated Macrophages with Anti-CSF-1R Antibody Reveals a Strategy for Cancer Therapy. Cancer Cell.

[99] Stafford J.H., Hirai T., Deng L., Chernikova S.B., Urata K., West B.L., Brown J.M. (2016). Colony stimulating factor 1 receptor inhibition delays recurrence of glioblastoma after radiation by altering myeloid cell recruitment and polarization. Neuro. Oncol..

[100] Pyonteck S.M., Akkari L., Schuhmacher A.J., Bowman R.L., Sevenich L., Quail D.F., Olson O.C., Quick M.L., Huse J.T., Teijeiro V. (2013). Articles CSF-1R inhibition alters macrophage polarization and blocks glioma progression. Nat. Med..

[101] DeNardo D.G., Brennan D.J., Rexhepaj E., Ruffell B., Shiao S.L., Madden S.F., Gallagher W.M., Wadhwani N., Keil S.D., Junaid S.A. (2011). Leukocyte Complexity Predicts Breast Cancer Survival and Functionally Regulates Response to Chemotherapy. Cancer Discov..

[102] Mitchem J.B., Brennan D.J., Knolhoff B.L., Belt B.A., Zhu Y., Sanford D.E., Belaygorod L., Carpenter D., Collins L., Piwnica-worms D. (2013). Targeting Tumor-In fi ltrating Macrophages Decreases Tumor-Initiating Cells, Relieves Immunosuppression, and Improves Chemotherapeutic Responses. Cancer Res..

[103] Zhu Y., Knolhoff B.L., Meyer M.A., Nywening T.M., West B.L., Luo J., Wang-Gillam A., Goedegebuure S.P., Linehan D.C., De Nardo D.G. (2014). CSF1/CSF1R blockade reprograms tumor-infiltrating macrophages and improves response to T-cell checkpoint immunotherapy in pancreatic cancer models. Cancer Res..

[104] Kumar V., Donthireddy L., Marvel D., Condamine T., Wang F., Lavilla-Alonso S., Hashimoto A., Vonteddu P., Behera R., Goins M.A. (2017). Cancer-Associated Fibroblasts Neutralize the Anti-tumor Effect of CSF1 Receptor Blockade by Inducing PMN-MDSC Infiltration of Tumors. Cancer Cell.

[105] Schmid M.C., Avraamides C.J., Dippold H.C., Franco I., Foubert P., Ellies L.G., Acevedo L.M., Manglicmot J.R.E., Song X., Wrasidlo W. (2011). Receptor Tyrosine Kinases and TLR/IL1Rs Unexpectedly Activate Myeloid Cell PI3Kγ, A Single Convergent Point Promoting Tumor Inflammation and Progression. Cancer Cell.

[106] De Henau O., Rausch M., Winkler D., Campesato L.F., Liu C., Cymerman D.H., Budhu S., Ghosh A., Pink M., Tchaicha J. (2016). Overcoming resistance to checkpoint blockade therapy by targeting PI3Kγ in myeloid cells. Nature.

[107] Kaneda M.M., Messer K.S., Ralainirina N., Li H., Leem C.J., Gorjestani S., Woo G., Nguyen A.V., Figueiredo C.C., Foubert P. (2016). PI3Kγ is a molecular switch that controls immune suppression. Nature.

[108] Pujade-Lauraine E., Guastalla J.P., Colombo N., Devillier P., François E., Fumoleau P., Monnier A., Nooy M., Mignot L., Bugat R. (1996). Intraperitoneal recombinant interferon gamma in ovarian cancer patients with residual disease at second-look laparotomy. J. Clin. Oncol..

[109] Beatty G.L., Chiorean E.G., Fishman M.P., Saboury B., Teitelbaum U.R., Sun W., Huhn R.D., Song W., Li D., Sharp L.L. (2011). CD40 Agonists Alter Tumor Stroma and Show Efficacy against Pancreatic Carcinoma in Mice and Humans. Science.

[110] Beatty G.L., Torigian D.A., Chiorean E.G., Saboury B., Brothers A., Alavi A., Troxel A.B., Sun W., Teitelbaum U.R., Vonderheide R.H. (2013). A Phase I Study of an Agonist CD40 Monoclonal Antibody (CP-870,893) in Combination with Gemcitabine in Patients with Advanced Pancreatic Ductal Adenocarcinoma. Clin. Cancer Res..

[111] Hoves S., Ooi C.-H., Wolter C., Sade H., Bissinger S., Schmittnaegel M., Ast O., Giusti A.M., Wartha K., Runza V. (2018). Rapid activation of tumor-associated macrophages boosts preexisting tumor immunity. J. Exp. Med..

[112] Gunderson A.J., Kaneda M.M., Tsujikawa T., Nguyen A.V., Affara N.I., Ruffell B., Gorjestani S., Liudahl S.M., Truitt M., Olson P. (2016). Bruton Tyrosine Kinase – Dependent Immune Cell Cross-talk Drives Pancreas Cancer. Cancer Discov..

[113] Sharma P., Hu-Lieskovan S., Wargo J.A., Ribas A. (2017). Primary, Adaptive, and Acquired Resistance to Cancer Immunotherapy. Cell.

[114] Gyori D., Lim E.L., Grant F.M., Spensberger D., Roychoudhuri R., Shuttleworth S.J., Okkenhaug K., Stephens L.R., Hawkins P.T. (2018). Compensation between CSF1R^+^ macrophages and Foxp3^+^ Treg cells drives resistance to tumor immunotherapy. JCI Insight.

